# Effects of Processing and Cooking on Physicochemical, Sensory, and Functional Properties of Food: Second Edition

**DOI:** 10.3390/foods14213601

**Published:** 2025-10-23

**Authors:** Sheng-Dun Lin

**Affiliations:** Department of Food Science and Technology, Hungkuang University, 1018, Section 6, Taiwan Boulevard, Shalu District, Taichung 433304, Taiwan; lin54@hk.edu.tw; Tel.: +886-4-26318652 (ext. 5038); Fax: +886-4-26319176

## 1. Introduction

With the growing consumer demand for healthy diets and functional foods, the selection and optimization of food processing technologies have become critical factors for preserving nutritional value, enhancing sensory attributes, and extending shelf life. Different processing and cooking methods exert significant effects on the physicochemical, sensory, and bioactive characteristics of foods, and can further improve the functionality and nutritional quality of traditional products. Moreover, agro-industrial by-products and natural plant resources represent valuable materials for the development of high–value-added foods. The central theme of this Special Issue, “Effects of Processing and Cooking on Physicochemical, Sensory, and Functional Properties of Food: Second Edition”, is to systematically explore how processing and cooking techniques influence food quality traits. It also highlights recent research advances in fortifying traditional foods with functional ingredients, valorizing agro-industrial by-products, and developing emerging processing technologies for functional food innovation. This Special Issue comprises 14 high-quality original research articles and two review papers. These studies cover the effects of processing and cooking on food quality characteristics, the enhancement of nutritional value in traditional foods through the incorporation of functional ingredients, the development and functional potential of agro-industrial by-products, and the applications and advances of emerging food processing technologies.

## 2. Influence of Processing and Cooking Technologies on Food Quality Characteristics

Dry heating, hydrothermal, and pressure–thermal treatments are widely employed physical approaches to modify cereal structure, improve functionality, and extend shelf life [1]. Extrusion cooking—an advanced thermo-mechanical process combining high temperature, pressure, and shear—has attracted significant interest due to its efficiency and versatility. During extrusion, the conformational rearrangements of gluten proteins (including disulfide bond disruption and reformation) directly influence product compactness and elasticity [2], while starch gelatinization is jointly regulated by moisture, temperature, shear intensity, and equipment configuration. Extrusion can also convert insoluble dietary fiber into soluble forms, enhancing nutritional and functional value [3]. Enzyme supplementation improves fiber composition, dough stability, and water absorption [4].

Lewko et al. (contribution 3) compared dry heating, hydrothermal, and low-temperature extrusion treatments for wheat flour modification, combining cellulase and xylanase in composite processing. The study showed that dry heating had limited compositional effects but markedly reduced baking quality (dough elasticity decreased to 44.83% and hardening index decreased to 1.48). Hydrothermal–enzyme-assisted treatment improved viscosity and stability, whereas steam application negatively affected protein structure. Extrusion–enzyme-assisted treatment significantly increased water absorption (by 55–67%) but decreased stability and gelatinization temperature, indicating starch reorganization. These findings provide theoretical guidance for controlling processing conditions to produce flours with specific rheological and baking properties, and suggest potential for clean-label bakery applications.

Tartary buckwheat (*Fagopyrum tataricum*), which contains over 70% starch (on a dry weight basis), represents a promising alternative starch source [5]. Extrusion increases its water absorption index and resistant starch (RS) content, and promotes the formation of slowly digestible starch [6]. Annealing enhances amylose content and hydration, while quercetin inhibits starch swelling and retrogradation [7,8]. Zheng et al. (contribution 5) analyzed and compared the physicochemical, rheological, and digestibility properties of autoclaved modified starch (ACB), autoclaved-pullulanase modified starch (ACPB), and native black buckwheat starch (NB). Modified starches showed higher short-range order, RS content, and water/oil absorption but lower syneresis. Both treatments induced shear-thinning behavior and low gel elasticity, indicating molecular chain degradation. ACPB exhibited greater resistance to enzymatic hydrolysis, confirming that pullulanase-mediated debranching effectively enhances digestibility resistance. This study underscores the potential of buckwheat starch modification in functional food development, particularly for snack, beverage, and pastry formulations.

The development of plant-based meat alternatives contributes to reducing animal fat consumption. Conventional full-factorial experimental designs for flavor optimization require numerous samples, whereas fractional factorial approaches, such as the Taguchi method, can significantly reduce sample size and improve efficiency [9]. To date, no study has applied the Taguchi method specifically to optimize plant-based meat flavoring processes. Sarkisyan et al. (contribution 6) employed this method to evaluate the effects of sugar type (fructose, glucose, xylose), sugar concentration (25–100 mM), and reaction temperature (140–160 °C) on Maillard reaction products and volatile compound formation. Temperature emerged as the most influential factor; notably, 25 mM xylose at 140 °C yielded the most pronounced meaty aroma and highest sensory acceptance. Volatile analyses identified aldehydes, ketones, and alcohols as key aroma contributors. These results demonstrate the utility of the Taguchi method for efficiently identifying optimal processing parameters in flavor design for plant-based products.

Tea polyphenols (TPs), particularly epigallocatechin gallate (EGCG), are major contributors to the bitterness and astringency of tea [10]. Proteins are often employed to reduce such undesirable sensory properties [11]. Date palm pollen (DPP), with a protein content exceeding 30% [12], represents a novel protein source. Mohamed et al. (contribution 8) investigated EGCG–DPP interactions. High-temperature extraction (80 °C) yielded more protein and improved thermal stability. Spectroscopic analysis revealed a 1:1 EGCG–DPP binding ratio, altering α-helix and β-sheet composition and enhancing hydrophobic and hydrogen bonding interactions. Sensory evaluation indicated that DPP–TP complexes from 80 °C extraction markedly reduced astringency. The study demonstrates that plant-derived proteins can form stable complexes with polyphenols, offering a strategy to mitigate bitterness while preserving tea flavor.

Fish sauce, a traditional condiment in Asian cuisines, derives its characteristic flavor from free amino acids and volatile compounds [13]. Xu et al. (contribution 9) examined how different cooking conditions (boiling for 10–60 min or stir-frying for 10–60 s) affect its quality. GC–MS and electronic nose analyses identified 38 volatiles, 10 of which (OAV ≥ 1) were key odorants. Heating significantly increased the total OAV (346.51 → 707.40) and free amino acid content (~4900 mg/100 mL), suggesting that temperature effectively modulates amino acid metabolism and flavor formation. The findings provide valuable insight into thermal processing control for optimizing fish sauce flavor quality.

American ginseng (*Panax quinquefolium* L.) contains saponins, polysaccharides, and phenolics with antioxidant, anti-inflammatory, antidiabetic, anticancer, and neuroprotective properties [14]. Li et al. (contribution 11) compared the differences in quality formation and properties of American ginseng by low-temperature softened hot-air drying (LTS-HAD), blanched hot-air drying (BL-HAD), steamed hot-air drying (ST-HAD), and vacuum freeze-drying (VFD). VFD best preserved color and microstructure, while LTS-HAD retained the highest ginsenoside Rg1 and Rb1 levels. ST-HAD samples exhibited reddish-brown coloration and the highest antioxidant activity, with the formation of new ginsenosides (Rg6, (S)/(R)-Rg3, Rk1, Rg5). Principal component analysis effectively discriminated samples based on drying method and bioactive compound composition. Collectively, VFD is suitable for preserving native quality, while ST-HAD offers potential for enhancing color and flavor attributes.

Metabolic syndrome (MetS) is characterized by co-occurring metabolic abnormalities—hyperglycemia or insulin resistance, dyslipidemia (high triglycerides and low HDL cholesterol), and hypertension—often accompanied by central obesity. Increasing evidence suggests that consumption of phytochemical-rich foods can improve metabolic function and reduce chronic disease risk. Tamarillo (*Solanum betaceum* Cav.) is rich in dietary fiber, vitamin C, potassium, polyphenols, anthocyanins, and carotenoids, while being low in fat and sodium [15]. Chen et al. (contribution 12) investigated the effects of ethanol concentration and simulated gastrointestinal digestion on the phytochemical profile and bioactivity of tamarillo parts. Extraction with 95% ethanol yielded the highest phenolic, anthocyanin, and carotenoid contents. Extracts exhibited strong antioxidant capacity and potent inhibitory activity against key MetS-related enzymes (pancreatic lipase, α-amylase, α-glucosidase, and ACE). Tamarillo thus shows promise as a functional food ingredient for MetS prevention. Future studies should focus on processing and storage strategies to preserve bioactivity and enhance practical applications.

Zucchini (*Cucurbita pepo* L.) is a good source of folate, vitamin C, and essential minerals such as potassium, magnesium, phosphorus, and iron [16]. However, the combined effects of cutting size and cooking method on its chemical and sensory properties remain underexplored. Abellán et al. (contribution 16) assessed different cube sizes (10 mm, 20 mm) and cooking methods (stir-frying, steaming, raw control) on zucchini quality. Larger cubes had higher dry matter and ash contents; stir-frying increased protein and antioxidant activity, while steaming preserved appearance and texture. Sensory results indicated higher preference for smaller cubes, and steamed samples showed the best appearance and color. Overall, cooking method exerted greater influence than cut size—stir-frying favored nutritional retention, whereas steaming enhanced sensory acceptance.

## 3. Enhancing the Nutritional Value of Traditional Foods Through the Incorporation of Functional Ingredients

Among the wide range of edible insects, *Tenebrio molitor* L. (yellow mealworm) has gained significant attention due to its balanced nutritional composition, high growth efficiency, and suitability for large-scale industrial production. It is considered one of the insect species with the greatest commercial potential and is the first insect approved by the European Union for human consumption. The larvae can be processed into frozen, dried, or powdered forms [17]. The nutrient profile of *T. molitor* can effectively address the limitations of cereal-based foods, particularly the deficiency in total protein and the imbalance of essential amino acids [18]. Draszanowska et al. (contribution 4) investigated the effects of *T. molitor* powder (TM) supplementation on the physicochemical, antioxidant, and sensory properties of oat cookies. Their findings revealed that TM addition significantly increased the protein and fat contents of the cookies, improved the n-6/n-3 fatty acid ratio, and enhanced the oleic acid content while reducing the proportion of palmitic acid. The cookies naturally contained relatively high levels of potassium and phosphorus, and TM supplementation further increased the concentrations of most minerals (except for manganese and sodium). Moreover, antioxidant capacity increased markedly with higher TM addition levels, primarily attributed to the presence of hydrophilic antioxidant compounds. Although TM incorporation reduced cookie lightness (L* value), the yellowness (b*), chroma, and hue angle were only slightly affected. The TM30 sample (containing 30% mealworm powder) exhibited a darker color and softer texture, which was consistent with sensory evaluation results. Overall, *T. molitor* serves as a promising functional ingredient for bakery reformulation, providing a sustainable and nutritionally enhanced alternative for food innovation and product redesign.

Although traditional foods are widely accepted, they often face challenges in meeting modern dietary expectations due to the presence of allergens or the absence of bioactive components. For instance, gluten proteins in wheat-based bread provide desirable viscoelastic properties but are the primary trigger of celiac disease. Gluten-free alternatives have thus been developed using rice, cassava, and legume flours [19]. *Linum usitatissimum* L. (flaxseed) is a nutrient-dense crop rich in bioactive compounds. Among them, hydrophobic cyclic peptides known as linusorbs (LOs, or cyclolinopeptides) exhibit multiple physiological activities, including the inhibition of osteoclast differentiation [20], antitumor effects against gastric cancer cells (SGC-7901) [21], and anti-inflammatory activity through the downregulation of the NF-κB signaling pathway [22]. Additionally, flaxseed is abundant in water-soluble polysaccharides and proteins [23]. Its protein and gum fractions can form natural coacervates that impart favorable texture, sensory properties, and rheological behavior to gluten-free products, making flaxseed a promising ingredient for functional gluten-free formulations. Shim et al. (contribution 7) developed gluten-free bread fortified with flaxseed meal and examined the stability of LOs in the flaxseed meal, flour mixtures, dough, and bread. The results showed that oxidized LOs remained stable during storage (up to four weeks), with the main compositional changes occurring during processing rather than storage. Reduced LOs were present at higher concentrations in the flaxseed meal and flour compared with the dough and bread, without a corresponding increase in the oxidized forms. Overall, flaxseed meal-fortified bread maintained satisfactory oxidative stability even under low-temperature conditions. This study is the first to assess the impact of the baking process on flaxseed cyclic peptide content and antioxidant activity, providing valuable insights for developing gluten-free bakery products with enhanced nutritional quality and compositional stability.

In the industrial production of California-style black olives, the fruits are treated with an alkaline solution and subjected to oxidation under aerated or non-aerated conditions to develop the characteristic dark color. They are subsequently packed in a brine containing sodium chloride, calcium chloride, and ferrous gluconate, and sterilized at 121–126 °C for 15–30 min to ensure microbial stability [24]. However, thermal processing may induce the formation of potentially toxic compounds such as heterocyclic aromatic amines and acrylamide [25], the latter of which is classified by the European Union as a carcinogenic contaminant subject to monitoring and regulation. *Cassia grandis*, a plant native to Central America, the Caribbean, and northern South America, produces fruits commonly known as Carao, which are rich in polyphenols, flavonoids, coumarins, saponins, and triterpenes [26]. This species exhibits antioxidant, anti-anemic, and analgesic activities, and has been successfully incorporated into dairy products such as yogurt to enhance nutritional and antioxidant properties without compromising sensory quality or lactic acid bacterial growth [27]. Montero-Fernández et al. (contribution 13) incorporated both fresh and freeze-dried Carao into California-style black olives to increase their mineral content, antioxidant potential, and total phenolic compounds, while evaluating its potential to reduce acrylamide formation. Their findings demonstrated that both forms of Carao significantly enhanced the iron content of the olives, along with concurrent increases in calcium, magnesium, and potassium levels. In terms of antioxidant capacity, the fresh Carao treatment yielded the greatest improvement, followed by the freeze-dried variant. Most notably, Carao supplementation significantly reduced acrylamide formation. These results indicate that *Cassia grandis* can serve as a natural functional additive to improve the nutritional value and safety of processed foods by simultaneously enhancing antioxidant activity and mitigating toxic compound formation.

## 4. Development and Functional Potential of Agro-Industrial By-Products

*Pumpkin* (*Cucurbita* spp.), a major member of the Cucurbitaceae family, is a nutrient-rich vegetable containing abundant β-carotene, dietary fiber, vitamins, and minerals, and has been reported to exhibit antidiabetic, antioxidant, anti-inflammatory, and hypolipidemic properties [28]. Pumpkin peels are generally regarded as processing waste; however, their protein, ash, lipid, and fiber contents are higher than those of the pulp [29]. Moreover, extracts from pumpkin peels display notable antibacterial and antioxidant activities, while peel-derived pectic dietary fibers can retard starch digestion and promote the growth of intestinal probiotics [30]. Alija et al. (contribution 10) investigated the effects of incorporating pumpkin peel powder (PS, 1–20%) into wheat flour for breadmaking. The results demonstrated that PS significantly influenced dough rheological properties and extended dough development time. Although increasing PS levels reduced bread loaf volume from 195.5 to 109.8 cm^3^, the antioxidant activity markedly improved, particularly in the crust (ABTS values increased from 2.37 to 10.08 μM TE/g DM). Additionally, total phenolic content and reducing sugar concentration increased proportionally with PS supplementation. These findings indicate that pumpkin peel powder, even at 20% substitution, maintains acceptable processing performance and presents great potential as a functional ingredient in bakery formulations.

During passion fruit (*Passiflora edulis* Sims) juice production, only the pulp is utilized, while approximately 70% of the peel and seeds are discarded, generating considerable agro-industrial waste [31]. Passion fruit by-products contain numerous bioactive compounds, including polyphenols, flavonoids, and carotenoids such as vitexin, resveratrol, chlorogenic acid, and β-carotene, which are associated with antioxidant, antihypertensive, hypoglycemic, and anti-obesity activities [32]. Passion fruit seeds are particularly rich in lipids—especially unsaturated fatty acids such as oleic and linoleic acids—as well as proteins and minerals [33], making them promising functional ingredients. Karaca Çelik et al. (contribution 14) incorporated passion fruit seed flour into cereal snack bar formulations. The inclusion of seed flour increased dietary fiber (from 4.17% to 5.66%), fat (from 15.02% to 19.63%), and antioxidant activity, primarily attributed to the presence of piceatannol, a resveratrol derivative. Although total phenolic content slightly decreased (from 90.11 to 65.37 mg GAE/100 g), the overall sensory acceptability remained high, and microbial safety was maintained during seven days of storage at 4 °C. These results highlight the feasibility of valorizing passion fruit seed by-products in functional snack formulations.

The industrial processing of *Ananas comosus* (L.) Merr. (pineapple) and *Citrus limon* (L.) Brum. (lemon) generates substantial quantities of peel by-products, which are rich in polyphenols and flavonoids exhibiting strong antioxidant potential. These bioactive compounds can mitigate oxidative stress and prevent chronic diseases [34,35]. Nevertheless, their utilization remains limited, typically confined to animal feed or composting. Lee et al. (contribution 15) employed a micronization technique combined with ultrasound-assisted extraction (UAE) using acidified ethanol solvents to enhance the recovery of antioxidant compounds from pineapple peel (PP) and lemon peel (LP). The optimal conditions—UAE for 30 min with 75% acidified ethanol—yielded the highest total phenolic contents at pH 5 (particle size 96.6 μm) for PP and at pH 4 (particle size 91.7 μm) for LP. In lemon peel extracts, hesperidin and eriocitrin concentrations reached 65.7 and 23.2 μg/mg, respectively. Contour and principal component analyses revealed that pH and extraction time had significant effects on antioxidant recovery efficiency, while Fourier-transform infrared (FTIR) spectroscopy and microscopic observations confirmed that smaller particle sizes and shorter drying times facilitated solute release. This study demonstrated that the integration of micronization with ultrasound-assisted extraction substantially improves the extraction efficiency of antioxidant constituents from fruit peels. Furthermore, the combination provides a data-driven, visualizable strategy for process optimization, offering valuable implications for sustainable bioprocessing and the development of functional bioactive ingredients.

## 5. Applications and Advances of Emerging Food Processing Technologies

Extrusion cooking is an advanced thermo-mechanical processing technology that integrates multiple unit operations, including conveying, mixing, heating, pressurization, and shearing. This high-efficiency, continuous, and controllable process has been widely applied in the production of human foods and animal feeds. Owing to the combined mechanical and thermal effects, extrusion cooking offers superior processing efficiency, nutrient retention, and environmental sustainability compared with conventional thermal processing. Natural medicinal and edible plants (NMEPs) refer to natural resources that possess both nutritional and therapeutic value and contribute to human health promotion. Their beneficial effects primarily derive from rich phytochemical constituents such as polysaccharides, flavonoids, saponins, and alkaloids [36,37]. These bioactive compounds form the biochemical foundation of traditional medicinal diets, nutraceuticals, and functional foods and play key roles in disease prevention and health maintenance. Traditional processing techniques—including cutting, drying, grinding, and solvent extraction—have been extensively utilized but remain limited in their ability to preserve bioactivity and enhance bioavailability. With the advancement of food technology, extrusion cooking has emerged as a promising technique for improving the processing efficiency and functional potential of NMEPs. Xu et al. (contribution 1) reviewed recent progress concerning the influence of extrusion parameters on the physicochemical properties, functional compounds (including total polyphenols, flavonoids, polysaccharides, and saponins), and pharmacological activities (such as antioxidant, hypoglycemic, hypolipidemic, and anti-inflammatory effects) of NMEPs. The review reported that the high temperature, pressure, and shear stress generated during extrusion disrupt plant cell walls and chemical bonds, facilitating the release and absorption of bioactive compounds. Although some heat-sensitive substances may partially degrade, the overall process enhances the availability and stability of active compounds. Given the significant variability in tissue structure and chemical composition among plant materials, extrusion parameters—such as barrel temperature, feed moisture content, screw speed, and screw configuration—must be optimized according to raw material characteristics. Rational process design can maximize the functional performance and ensure product consistency. Overall, extrusion cooking provides an innovative and theoretically grounded approach for the deep processing and value enhancement of NMEPs, offering a promising pathway toward the development of novel functional foods with combined nutritional and pharmacological benefits.

Traditional thermal processing remains the most common technique for plant-based fruit and vegetable products, primarily aimed at suppressing microbial growth and delaying quality deterioration. However, this approach often results in textural softening and degradation of heat-labile nutrients, such as vitamins, thereby reducing consumer acceptability. High-pressure processing (HPP) has recently emerged as a non-thermal sterilization technology capable of achieving microbial safety and quality preservation without the application of high heat. HPP employs a liquid medium—typically water—to uniformly transmit hydrostatic pressure (up to 1500 MPa) to sealed foods, effectively inactivating microorganisms and extending shelf life under commercial conditions of 400–600 MPa [38,39]. The main control parameters of HPP include pressure level, holding time, processing temperature, and adiabatic heating caused by compression (approximately 2–3 °C increase per 100 MPa) [38]. Compared with conventional pasteurization, HPP causes markedly less thermal degradation, allowing for improved retention of natural color, aroma, and texture. With increasing consumer demand for fresh, minimally processed, and plant-based foods, the application of HPP has expanded to the processing of whole fruits and vegetables (WFV). Textural stability is a critical determinant of WFV quality; thus, a comprehensive understanding of how HPP influences both enzymatic and non-enzymatic texture-related factors is essential for process optimization and industrial application. Non-enzymatic factors include turgor pressure, cell membrane integrity, intercellular adhesion, β-elimination reactions, and acid hydrolysis, while enzymatic regulation involves enzymes such as pectin methylesterase (PME), polygalacturonase (PG), cellulase, peroxidase, and β-galactosidase [40]. Heaney et al. (contribution 2) systematically analyzed previous studies to elucidate the effects of HPP parameters (pressure, holding time, and temperature) on the textural characteristics of non-beverage fruits and vegetables. Their review emphasized enzymatic texture-modulating mechanisms—such as PME and PG activities and pectin esterification—as well as non-enzymatic physical attributes, including hardness, cohesiveness, springiness, chewiness, and resilience. Moreover, the review examined texture recovery during storage, the reinforcing effects of combining HPP with calcium ions or PME pre-treatment, and the influence of isoenzyme profiles and substrate microenvironments on texture stability. Collectively, the findings demonstrate that HPP, whether applied independently or in combination with mild thermal treatments, holds great potential for maintaining or even improving texture quality during WFV processing. The technology thus represents a sustainable alternative to conventional thermal processing and provides a scientific foundation and practical pathway for innovation and quality enhancement in the plant-based food industry.

## 6. Conclusions

Food processing and cooking technologies exert profound effects on the physical, chemical, bioactive, and sensory characteristics of food products. Conventional thermal treatments, extrusion cooking, and enzymatic modification can improve the structural, functional, and nutritional properties of cereal and plant proteins. Meanwhile, natural plants and agro-industrial by-products serve as valuable resources for functional food development. Emerging technologies such as extrusion cooking and high-pressure processing effectively preserve bioactive compounds, enhance texture, and improve overall product functionality, demonstrating significant potential for advancing the health-oriented and sustainable food sectors. Looking ahead, the development of functional and health-promoting foods requires precise integration of raw material characteristics with appropriate processing technologies. Optimizing processing parameters and formulation design will be essential to balance nutritional fortification, sensory quality, and product safety. Furthermore, the integration of waste reutilization strategies with innovative technologies offers promising prospects for sustainability and value creation in the food industry. Future research should focus on elucidating the stability, metabolic activity, and consumer acceptability of functional compounds under combined processing conditions to support industrial implementation and sustainable growth of the functional food market.
